# Artistic Robotic Arm: Drawing Portraits on Physical Canvas under 80 Seconds

**DOI:** 10.3390/s23125589

**Published:** 2023-06-14

**Authors:** Shady Nasrat, Taewoong Kang, Jinwoo Park, Joonyoung Kim, Seung-Joon Yi

**Affiliations:** Electrical Engineering Department, Pusan National University, Busan 46241, Republic of Korea

**Keywords:** robotic portrait drawing, calligraphy pen, CycleGAN

## Abstract

In recent years, the field of robotic portrait drawing has garnered considerable interest, as evidenced by the growing number of researchers focusing on either the speed or quality of the output drawing. However, the pursuit of either speed or quality alone has resulted in a trade-off between the two objectives. Therefore, in this paper, we propose a new approach that combines both objectives by leveraging advanced machine learning techniques and a variable line width Chinese calligraphy pen. Our proposed system emulates the human drawing process, which entails planning the sketch and creating it on the canvas, thus providing a realistic and high-quality output. One of the main challenges in portrait drawing is preserving the facial features, such as the eyes, mouth, nose, and hair, which are crucial for capturing the essence of a person. To overcome this challenge, we employ CycleGAN, a powerful technique that retains important facial details while transferring the visualized sketch onto the canvas. Moreover, we introduce the Drawing Motion Generation and Robot Motion Control Modules to transfer the visualized sketch onto a physical canvas. These modules enable our system to create high-quality portraits within seconds, surpassing existing methods in terms of both time efficiency and detail quality. Our proposed system was evaluated through extensive real-life experiments and showcased at the RoboWorld 2022 exhibition. During the exhibition, our system drew portraits of more than 40 visitors, yielding a survey outcome with a satisfaction rate of 95%. This result indicates the effectiveness of our approach in creating high-quality portraits that are not only visually pleasing but also accurate.

## 1. Introduction

Robotic portrait drawing has attracted considerable attention from researchers due to its fascinating challenges in both technical and creative domains. With the increasing adoption of collaborative robots, numerous robotic portrait drawing systems have emerged. However, achieving a balance between the quality of the output drawing and the efficiency of the drawing process has been a significant hurdle. Many approaches have prioritized either producing highly detailed drawings, which can be time consuming, or simplifying facial features to complete the drawing quickly.

In this paper, we propose a novel approach that combines these objectives by leveraging advanced machine learning techniques and a variable line width Chinese calligraphy pen. The proposed approach adeptly balances the trade-off between drawing speed and output quality, thereby offering a unique solution. Our system emulates the human drawing process, involving sketch planning and creation on the canvas. Preserving facial features poses a major challenge in portrait drawing, which our system overcomes by utilizing CycleGAN, a technique that retains essential details such as the eyes, mouth, nose, and hair. The proposed system employs a human keypoint detection algorithm to identify and crop the dominant human face from a video feed. Subsequently, the CycleGAN algorithm is applied to perform style transfer, transforming the image into a black-and-white sketch. From this sketch image, line extraction and path optimization algorithms are utilized to generate optimized waypoints to guide a robotic arm during the physical tracing process. Finally, a six DOF robotic arm equipped with a Chinese calligraphy pen is employed to draw the portrait, dynamically adjusting the pen pressure to vary the stroke width.

To assess the effectiveness of our system, we conducted extensive real-world experiments involving various volunteer groups. The results demonstrate that our system can produce high-quality portrait drawings with an average drawing time of 80 s while preserving most of the facial details. Furthermore, the system was showcased to the general public at the RoboWorld 2022 exhibition, where it successfully drew portraits of over 40 visitors, achieving a satisfaction rate of 95%. In contrast with existing methods, our proposed system offers the following key contributions:

- Drawing with variable stroke widths utilizing a Chinese calligraphy pen.
- Style transfer to variable-width black and white pen drawings using CycleGAN.
- Effectively balancing between time consumption and portrait quality.
- High level of satisfaction from the public demonstration volunteers.

The remainder of this paper is organized as follows: Section 2 provides a review of previous approaches to robotic portrait drawing systems, categorizing them into two broad categories and highlighting the advantages of our proposed system. Section 3 offers detailed insights into each of the four modules comprising our system. Section 4 presents the hardware setup, including the utilization of a Chinese calligraphy pen to achieve variable stroke widths. Section 5 presents the experimental results, including the public demonstration conducted at the RoboWorld 2022 exhibition. Finally, we conclude by discussing the future directions of this research in Section 6.

## 2. Related Work

The utilization of robotic systems for drawing and painting has gained significant attention among researchers in recent years, as demonstrated in various papers. For instance, ref. [1] presents a new approach to designing a controller for dual-arm manipulation that can be used for various tasks such as drawing. Collaborative painting robots have been developed in [2], where a digital twin framework was used to simulate the painting process and estimate the paint result before real execution, reducing set costs, waste, and time. In [3], a generative adversarial network, a co-robotic arm and a 5-year-old child were combined to establish a visual–mental–physical circuit for communication between the human and non-human actors. Furthermore, inverse kinematic models were developed in [4] using an artificial neural network method to control the movement of a three DOF arm drawing robot. GeomBot, a drawing robot that combines Scratch and Papert’s drawing turtle, was designed in [5] for geometry activities for primary school classes. A three DOF robotic arm made from LEGO NXT bricks was used for drawing on paper in [6], and was deemed suitable for educational projects on robotics and robot programming. Additionally, behavior-based control methods were used for brush drawing in [7], and a drawing robot system that can draw any picture automatically was proposed in [8]. Finally, the power of eye tracking as a powerful tool for assistive technologies was presented in [9,10], where a robot arm controlled by eye tracking was demonstrated. It is suggested that gaze-based decoding may become one of the most efficient ways to interface with robotic actuators. The KUKA Agilus industrial robot arm was used in [11] as an interface platform between the robot and personal computers, with two tools designed for collecting logging data and drawing image files. including the reduction of complexity and computational burden in flexible multibody systems [12], dynamic reduction algorithms for flexible mechanisms [12], and the development of integral robust control algorithms for uncertain nonlinear systems [13]. Additionally, several research studies have also introduced neuroadaptive learning algorithms for robust control in constrained nonlinear systems and robotic painting systems, including studies such as [14,15,16,17,18,19,20].

However, the use of machine learning in robotic portrait drawing poses several challenges. The primary difficulties include preserving fine details and achieving short drawing times. In previous methods, various drawing tools such as paint brushes [21,22,23,24] have been employed, but they often struggle to capture fine details and they are also time consuming. For example, [23] utilized paint brush techniques to create highly detailed portraits, but this required a large number of strokes, resulting in lengthy drawing times; a single portrait took 17 h to complete a drawing with 9000 strokes. Other methods, such as those that use pens or pencils [10,25,26,27,28], are also time consuming and may fail to preserve facial details in the drawings.

To overcome these challenges and improve the efficiency of robotic portrait drawing, researchers have increasingly turned to machine learning techniques. For example, Gao et al. in [29] employed GAN-based style transfer to reduce the number of strokes required for drawing sketches, resulting in shorter drawing times. However, this approach resulted in the simplification of portrait drawings, sacrificing the preservation of facial details. Similarly, Tianying et al. in [25] used GAN-based style transfer to transform a target face image into a simplified cartoon character, which reduced the average drawing time to 43.2 s. While these techniques may be efficient, they often lack the ability to produce highly detailed drawings.

To provide a comprehensive understanding of our approach, we have included a detailed comparison section that compares our system to other research papers in the field of robotic portrait drawing. The comparison includes various aspects such as the robotic arm’s degree of freedom, drawing time, and the drawing tool used. We divided the comparison into two tables to provide a clear overview. The first Table 1 presents a comparison of papers that utilize pens, pencils, or markers as drawing tools. These methods are known for their time efficiency but have limitations when it comes to preserving fine details in the drawings. On the other hand, the second half of Table 1 compares research papers that utilize calligraphy pens or paint brushes as drawing tools. These methods are known for their ability to produce highly detailed drawings, but they can be time consuming.

In summary, our proposed system provides a solution to the challenges faced by previous methods in robotic portrait drawing. By utilizing advanced machine learning techniques and a path optimization algorithm, we can achieve efficient and detailed portraits. The comparison section provides an overview of the current state of the art in the field and highlights the advantages of our approach.

## 3. Proposed Methods

**Figure 1:**
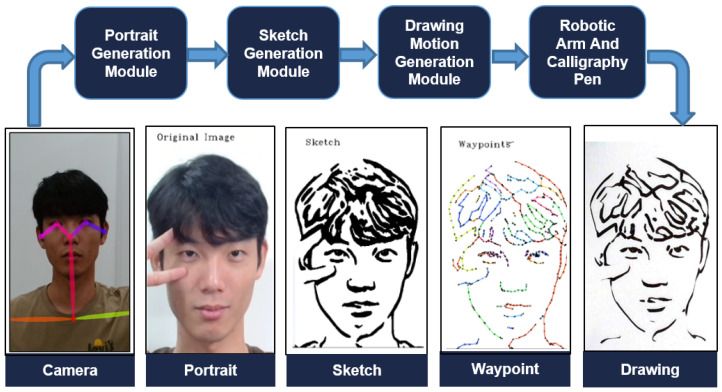
System architecture and output flow. The diagram illustrates the flow of input portrait image, processed sketch image, generated waypoints, robotic arm motion data, and final drawing output.

The proposed system is designed to achieve the objective of generating high-quality portraits on a physical canvas using a four-module approach. The system consists of four main modules seen in Figure 1. The first module, the Portrait Generation Module, is responsible for capturing high-quality RGB portrait images from the camera, which serves as the foundation for the subsequent steps of the process. This module’s accuracy is essential to ensure that the overall process is performed with the highest level of precision. The second module, the Sketch Generation Module, employs the CycleGAN to create the desired sketches from the RGB portrait images. This module plays a significant role in converting the captured images into sketches that can be used as a reference for the physical canvas. The third module, the Drawing Motion Generation Module, is responsible for converting the sketch images into traceable navigational waypoints. These waypoints are then followed by the Robot Motion Control Module on the physical canvas, enabling smooth and precise execution of the process. The success of the entire project relies heavily on the accuracy and reliability of this module.

To gain a deeper understanding of each module’s related work, separate sections will be dedicated to discussing their functionality, strengths, and limitations. Through this comprehensive analysis, the proposed system’s performance and potential for future improvements can be better evaluated.

### 3.1. Portrait Generation Module

The incorporation of the OpenPose algorithm [33] is an obvious asset to our module, as it allows for the efficient and accurate cropping of human faces or regions of interest (ROI) from camera frames. This algorithm is predicated on the utilization of a convolutional neural network (CNN), which has been trained to identify and locate critical points on the human body, including the shoulders, elbows and wrists. By detecting these key points, the OpenPose algorithm is able to precisely crop a person’s face from the frame, even when the face is partially obscured or when the person is positioned at an uncommon angle. Moreover, the algorithm’s capacity for real-time applications makes it an ideal choice for this purpose.

**Figure 2:**
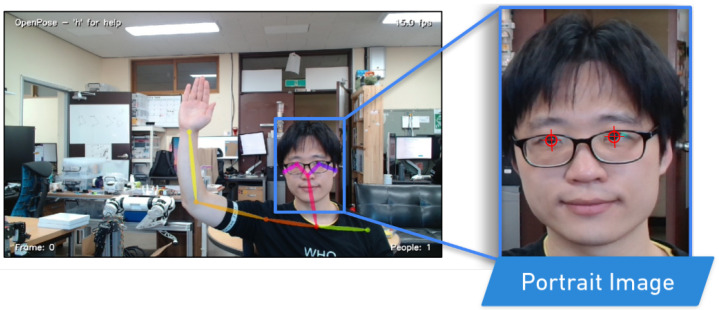
Extracting portrait image and its eye positions from RGB camera input utilizing human keypoint detection algorithm.

Additionally, apart from its ability to crop images, we have implemented the OpenPose algorithm to retrieve the precise position of the eyes seen in Figure 2. This extracted information concerning eye position is then utilized in the path optimization module, which is responsible for generating the optimal path for the robotic arm to follow during the eye-drawing phase. This integrated approach streamlines the eye-drawing process, as it allows for a more efficient and accurate execution of the desired outcome.

### 3.2. Sketch Generation Module

Our study introduces a novel system for generating sketch-style avatars from real face images, which utilizes the CycleGAN [34] to learn a mapping between the domains of real faces ($X_{real}$) and sketch-style avatars (*Y*). The mapping is designed to preserve the consistency of essential facial features, such as haircuts, face shapes, and eye shapes, while learning the relationship between the two domains. This enables the system to generate high-quality portrait sketches that accurately reflect the real face images, without the need for any labeled data or supervision.

A significant advantage of the CycleGAN is its ability to handle a diverse range of facial features and styles, making it an effective tool for generating sketch-style avatars that preserve the consistency of the features, even when the real faces and avatars have significant differences in appearance. The flexibility of the CycleGAN allows for the generation of sketches that are faithful representations of the real faces, which makes the system ideal for use in various applications.

**Figure 3:**
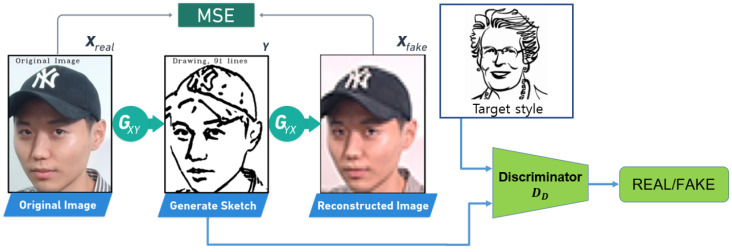
The Sketch Generation Module. The system is composed of three main components: the training dataset of real faces ($X_{real}$), the training dataset of generated faces ($X_{fake}$) and the generated sketches (*Y*). The Generator network $G_{XY}$ converts real face images ($X_{real}$) into sketch images (*Y*), while the inverse Generator network $G_{YX}$ reconstructs the sketch images (*Y*) back into generated face images ($X_{fake}$).

CycleGAN can be used in many situations due to its unsupervised approach, which enables it to be highly flexible and adaptable. The structure of the CycleGAN is depicted in Figure 3, where $X_{real}$ denotes the real faces used for training, $X_{fake}$ represents the generated faces, and *Y* represents the generated sketches. To convert face images to sketch images, we employ a generator network $G_{XY}$, while a generator network $G_{YX}$ is utilized to reconstruct sketch images to face images. The training of $G_{XY}$ and $G_{YX}$ is regulated using mean square error MSE.

To ensure the accuracy of the generated sketches, we use a drawing discriminator $D_{D}$, which discriminates between the generated portrait line drawings and the real ones. $D_{D}$ is used to enforce the existence of essential facial features in the generated drawing. Moreover, we employ three local discriminators, $D_{ln}$, $D_{le}$, and $D_{ll}$, that focus on discriminating nose drawing, eye drawing, and lip drawing, respectively. The inputs to these local discriminators are masked drawings, where masks are obtained from a face parsing network. The drawing discriminator $D_{D}$ comprises *D*, $D_{ln}$, $D_{le}$, and $D_{ll}$, which work together to ensure the accuracy of the generated sketches. Additionally, the use of the drawing discriminator and local discriminators ensures that the generated sketches preserve essential facial features accurately, making them ideal for use in various facial recognition and image editing applications.

### 3.3. Drawing Motion Generation Module

The primary objective of this module is to develop an efficient and accurate method for extracting generated sketch lines through the implementation of advanced machine learning techniques and optimized line extraction. In order to achieve this goal, we have concentrated on extracting the minimum number of lines necessary to retain all facial features. The extracted lines are then converted into waypoint data that can be utilized by the robot motion module. By utilizing this approach, we aim to create a highly effective method for generating portrait sketches that provides both precision and speed.

#### 3.3.1. Skeleton Extraction

In order to generate smooth and accurate drawing motions, it is necessary to employ a series of preprocessing steps on the sketch. One such crucial step is the application of morphological transformation to extract motion waypoints from the sketch. The extraction process involves simplifying the sketch into a structure of lines, which can be easily processed. To achieve this, two different morphological transform algorithms are used for skeletonization: the opening algorithm and the closing algorithm.

The opening algorithm is primarily used to extract the majority of the sketch lines. This algorithm involves performing erosion followed by dilation, which helps to smooth pixel edges and eliminate isolated pixels. This step significantly simplifies the sketch structure and allows for better feature extraction in the subsequent steps. On the other hand, the closing algorithm is primarily employed to improve the accuracy of the system for eye sketches. This algorithm involves performing dilation followed by erosion, which fills small holes and provides better details. This step helps to produce more natural-looking portraits.

#### 3.3.2. Lines Extraction

The pixel-to-pixel Algorithm 1 was employed prior to extracting lines from a sketch image. This analysis was conducted on a down-sampled version of the cropped image, where the width was reduced to 400 pixels. This technique entails a search for the nearest pixel to the current pixel and appending it to a line array if it is connected. Pixels that are not connected are saved to a new line array. The pixel-to-pixel algorithm is highly effective and accurate when it comes to extracting lines from sketches, enabling their further processing in the system. The method facilitates the extraction of crucial details and features of the sketch, including facial features, while minimizing the number of lines and optimizing the processing time. It is a crucial step in the creation of a high-quality portrait sketch.

By implementing the pixel-to-pixel algorithm, we were able to extract important features and details from the sketch image accurately and efficiently. The algorithm’s process of searching for connected pixels and adding them to a line array enabled the isolation of key features of the sketch, which could be processed further in the system. Additionally, the algorithm’s ability to identify non-connected pixels allowed us to save them to a new line array, ensuring that no important features or details were lost in the extraction process. This approach enabled us to extract critical facial features from the sketch image with minimal noise, resulting in a high-quality portrait sketch.

#### 3.3.3. Lines Clustering and Waypoints Generation

In this proposed system, we have implemented a line clustering algorithm that categorizes lines based on their spatial proximity to one another with the aim of minimizing the number of lines that need to be drawn by the robotic arm. By recognizing clusters of lines that are situated in close proximity, we are able to merge them into a single line. This approach resulted in a reduction in the average number of lines drawn to 49%, leading to more efficient and faster production of high-quality drawings. As an illustration, the lowest line count recorded was reduced to 45 lines, resulting in a drawing time of 75 s.

Once the lines have been clustered, we use a waypoint generation algorithm to extract the necessary information from the sketch image. We have set the waypoint generation algorithm buffer to 250 waypoints per line, which allows us to maintain a high level of precision while also optimizing the drawing process. The waypoints generated by this algorithm not only contain information about the path that the arm should follow, but also hold information about the thickness of the lines being drawn.

After the waypoints have been generated, they are passed to the robot motion control module, which uses them to guide the arm along the desired path. This allows the arm to smoothly trace the lines in the sketch and produce high-quality drawings in a timely manner.
**Algorithm 1:** Extract Line: *Pixel to Pixel*
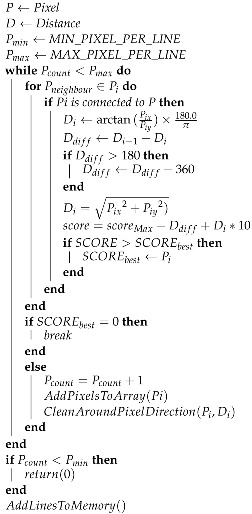


#### 3.3.4. Eye Handling

**Version 1: Direct Sketch Features Extraction**

**Figure 4:**
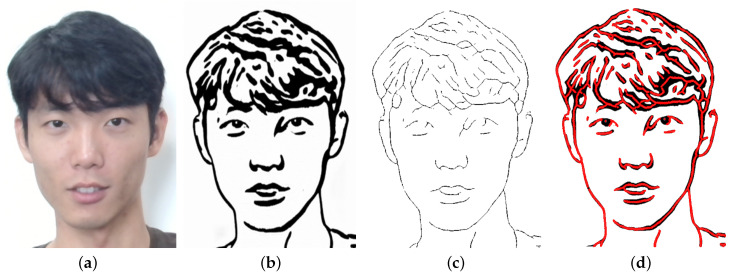
Visualization of skeleton extraction process. (**a**) original image; (**b**) sketch image; (**c**) extracted skeleton; (**d**) sketch image overlaid by extracted skeleton.

In our initial attempt at extracting lines from sketch images, we utilized the Closing Algorithm seen in Figure 4. However, due to the complex structure of the eye lines and pupils in the sketch, this approach was not able to effectively extract these lines, resulting in the loss of eye details and the smoothing out of features. As can be seen in the extracted lines in Figure 4c, the Closing Algorithm was unable to accurately capture the nuances of the eye sketch, leading to a significant reduction in eye detail. In order to address this issue, we focused on developing alternative approaches in subsequent versions of the system.

**Version 2: Replacing eye pupil sketch with circle**

To address the issue of detail loss in the eye sketch, we implemented a first approach that involved replacing the eye pupil sketch with a circle. This process involved the use of the Open Pose algorithm to identify the approximate position of the eyes in the sketch, followed by the clearing of the sketch at these positions. The remaining lines were then extracted and processed to generate waypoints, with additional waypoints in the form of circles added at the positions of the eye pupils. While this approach successfully preserved the details of the eye sketch, it was sensitive to errors in eye position recognition, leading to a less natural appearance in the final drawing compared to the original sketch. To address this issue, we introduced a third version of the system.

**Version 3: Eye lines extraction**

**Figure 5:**
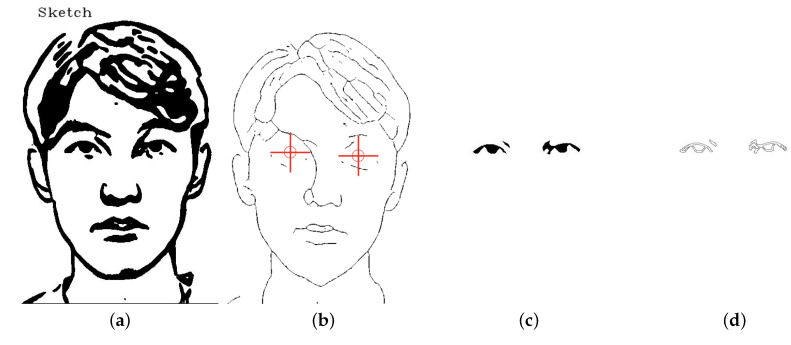
Visualization of eye area handling process. (**a**) Sketch image; (**b**) skeleton image; (**c**) extracted eye; (**d**) eye edges.

**Figure 6:**
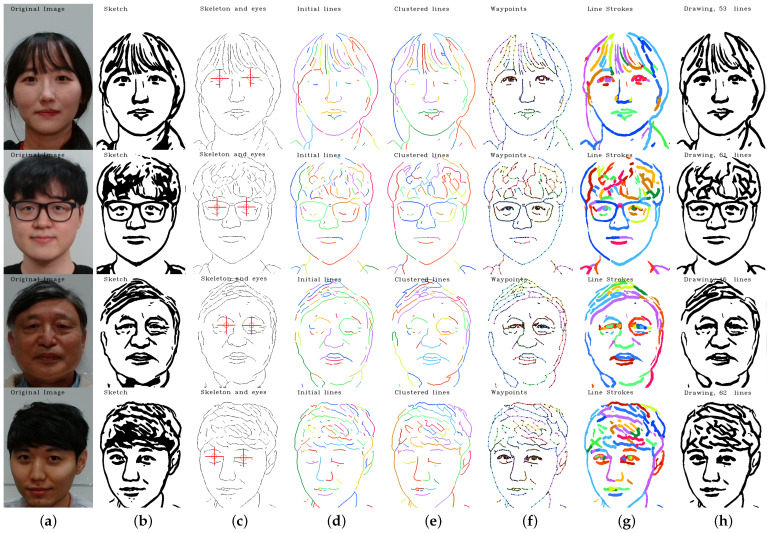
Visualization of the sketch image processing steps in different drawing versions, including (**a**) portrait image; (**b**) sketch image; (**c**) skeleton and eye triggers; (**d**) initial lines excluding eye lines; (**e**) clustered lines; (**f**) generated waypoints; (**g**) line strokes; (**h**) predicted drawing.

**Figure 7:**
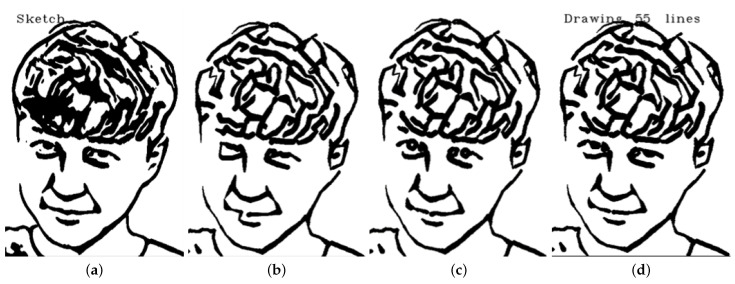
Comparison of the sketch image and the predicted drawings generated by different versions of our system, showing the progression from sketch image to final output. (**a**) sketch image; (**b**) version 1; (**c**) version 2; (**d**) version 3.

To further improve the accuracy and natural appearance of the eye sketch, we implemented an approach that involved separating the eye sketch from the rest of the image and processing them separately. We used the Opening algorithm for the eye sketch seen in Figure 5 and the Closing algorithm for the rest of the image. This approach allowed us to accurately capture the details of the eye without smoothing out or filling in any features, as was the case with the Closing algorithm in the first version. As a result, the final sketch had a more natural appearance and was able to accurately preserve the details of the eye as seen in Figure 6. This approach proved to be the most stable and reliable method for producing accurate eye drawings. Refer to Figure 7 for a comparison between the 3 versions.

### 3.4. Robot Motion Control Module

We implemented a path optimization algorithm and a communication system, using ROS, to improve the efficiency and precision of the robotic arm’s portrait drawing process. The path optimization algorithm calculates the most efficient path for the arm to reach the next starting point, minimizing unnecessary movements and reducing the drawing time. To ensure that the robotic arm had enough information to accurately draw a single line, we set the waypoint navigational buffer to 250 points, as determined by the previously discussed waypoint generation algorithm. The communication system, using ROS, allowed for smooth and precise control of the robotic arm during the drawing process. Together, these techniques resulted in a smooth and efficient drawing process, producing high-quality portrait sketches in a timely manner.

## 4. Hardware Setup

**Figure 8:**
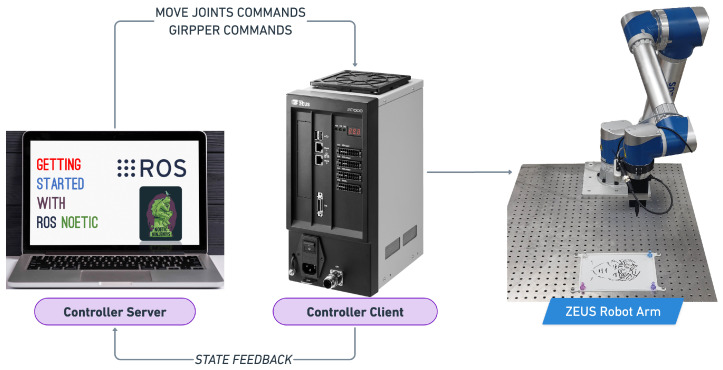
Flow chart showing communication between controller server and client with the robotic arm.

Our hardware setup consists of a robotic arm mounted on a table as seen in Figure 8, equipped with a custom-designed pen gripper that is capable of securely holding calligraphy brush pens in a fixed position. The pen gripper is specifically designed to prevent the pens from slipping, ensuring that the drawings produced are of high quality and accuracy. Additionally, we have designed a 3D printed canvas pad that holds A5 paper steady and keeps it in place during the drawing process. To ensure that the paper remains firmly in place, we have incorporated the use of magnets on the canvas pad. The selection of the A5 canvas size serves multiple practical purposes, with the most prominent being the optimal line thickness achievable with the Chinese calligraphy pen. Given the pen’s limitation of producing lines with a maximum thickness of 2.25 mm, utilizing a larger canvas would diminish the impact of adjusting line thicknesses. Furthermore, we utilized an MSI laptop as the controller server for computational tasks, facilitating efficient processing and coordination of the robotic arm’s movements. The specifications of the MSI laptop used are provided in Table 2.

### 4.1. Robotic Arm

The ZEUS robotic arm [35], is an industrial automation device with six axes of motion designed to handle various tasks in manufacturing, assembly, and material handling. Its cutting-edge features include a pen gripper attachment that enables precise tool manipulation, making it ideal for tasks demanding intricate and accurate operations. The ZEUS arm was primarily employed in the RoboWorld 2022 competition, where it was provided to participating teams by a sponsoring company. Given that the robotic arm we utilized shares similar workspace and maximum linear speed specifications with the commonly used UR5 robotic arm, we believe that the current system’s performance can be effortlessly replicated with other comparable robotic arms.

### 4.2. Pen Gripper and Canvas Pad

**Figure 9:**
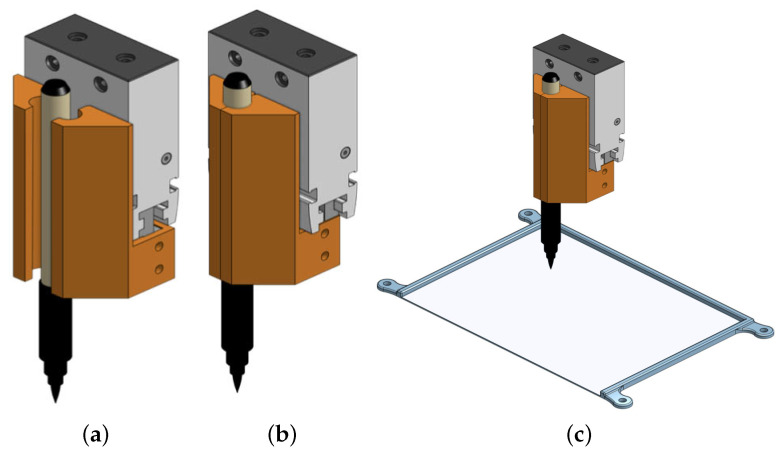
3D models of gripper and canvas pad. (**a**) open gripper; (**b**) closed gripper; (**c**) canvas pad.

This gripper is specifically designed to securely hold calligraphy brush pens in a fixed position, preventing them from slipping during the drawing process seen in Figure 9. By utilizing a 3D printing process, we are able to tailor the design of the gripper to the specific requirements of our system, ensuring optimal performance and reliability.

In addition to the custom-designed gripper, we have also implemented a 3D printed canvas pad to hold the A5 paper steady and keep it in place during the drawing process. By 3D printing the canvas pad, we are able to customize its shape and size to perfectly fit the dimensions of the paper, ensuring that it remains firmly in place throughout the drawing process. The use of 3D printing technology allows us to achieve a high level of precision and customization in the design of both the gripper and the canvas pad.

### 4.3. Calligraphy Pen

**Figure 10:**
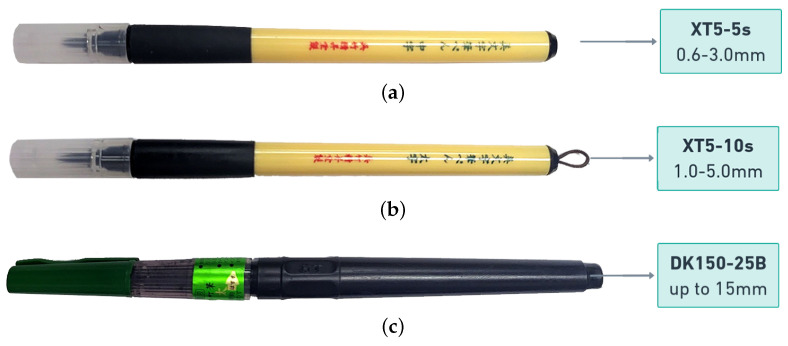
Showing 3 Calligraphy Brush Pens used in calligraphy pen experiment. (**a**) XT3–5s; (**b**) XT3–10s; (**c**) DK150–25B.

In our experiment, we took great care in selecting the appropriate calligraphy pens for use. We used extensive testing to find out which pens would best serve our needs and produce the desired results. We utilized Kuratake Bimoji Calligraphy Brush pens XT3-5s, XT3-10s and DK150-25B as suitable options for our needs. We used the XT3-5s producing 0.6–3.0 mm line thickness (refer to Figure 10a), the XT5-10s producing 1.0–5.0 mm line thickness (refer to Figure 10b), and the DK150-25B with up to 15.0 mm width (refer to Figure 10c).

**Figure 11:**
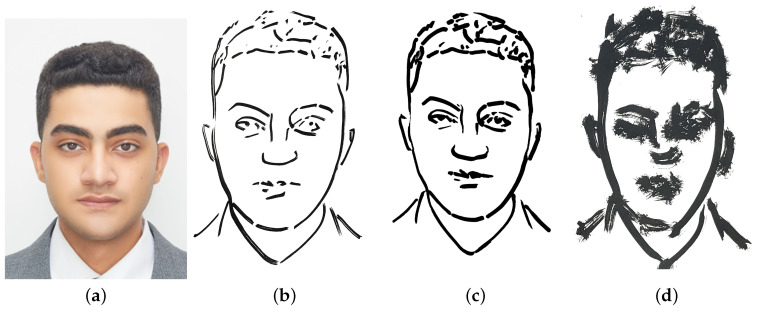
Comparison of drawings created using three different Chinese calligraphy pens. (**a**) Original; (**b**) XT5-5s; (**c**) XT5-10s; (**d**) DK150-25B.

Through analysis, we found that the DK150-25B with up to 15.0 mm width, which has a tip made of horse tail, produced blot lines when applied to thick lines, as shown in Figure 11c. As a result, we decided not to use it in our experiment. Additionally, we also evaluated the XT5-5s. Although it produced blot lines, it did not demonstrate the same level of performance and line thickness as the XT5-10s as shown in Figure 11b. Ultimately, we determined that the Extra Large (XT5-10s) with a 1.0 to 5.0 mm line thickness was the most appropriate choice for our primary pen. This decision was based on its ability to produce thick lines with a high degree of precision, without any deformation or blotting.

**Figure 12:**
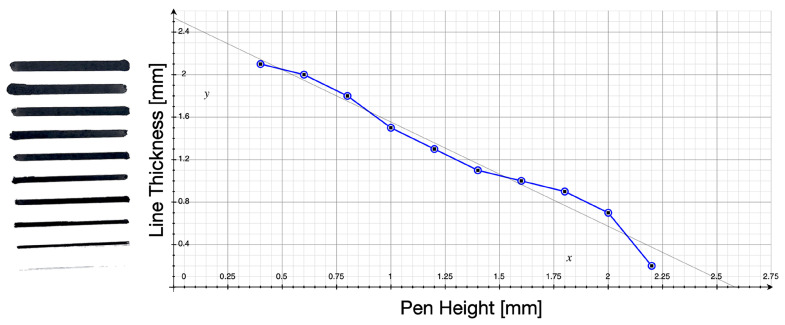
Pen calibration process. Test drawing image and Relation between the line thickness and pen height.

In order to accurately and consistently reproduce the desired line thickness in our robotic portrait drawing system, we conducted a series of calibration tests to determine the relationship between pen height and line thickness. Using calligraphy brush pens, we measured the resulting line thickness at different heights above the canvas. The results of these tests were plotted on a correlation graph, as shown in Figure 12, which showed that a line thickness range of 0.1 mm to 2.25 mm was achievable using our system. This information was then used to fine-tune the movement of the robotic arm and ensure that the desired line thickness was consistently achieved in our portrait drawings.

## 5. Experimental Results

The computational average time per sketch is a crucial aspect with which to evaluate the efficiency and practicality of the proposed robotic portrait drawing system. Table 3 presents the results of the experiments conducted, demonstrating the average time taken by the system to generate each sketch.

### 5.1. Lab Experiment

**Figure 13:**
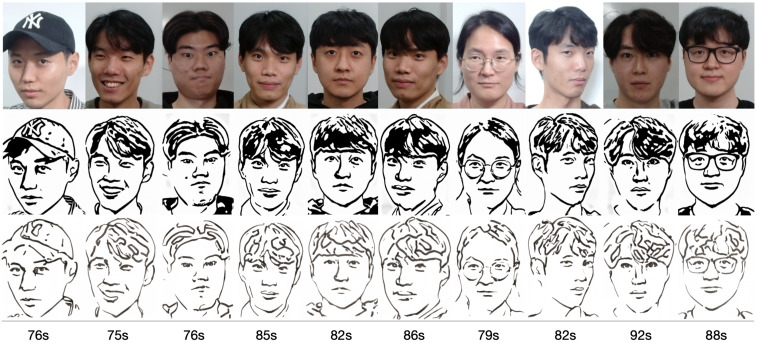
Examples of drawing time experiments, illustrating the progression from the original image to the sketch image to the final drawings on the physical canvas.

In order to assess the efficacy and precision of our system, a series of experiments were carried out. These experiments involved the use of our robotic arm to create portraits of a diverse range of volunteers, as illustrated in Figure 13. The time taken to produce each portrait was recorded and averaged to be approximately 1 min and 20 s. These experiments were conducted as a segment of the highly esteemed ZEUS competition. Our system was demonstrated to be highly efficient and precise at generating high-quality portrait sketches within a relatively short period of time.

### 5.2. Public Demonstration

**Figure 14:**
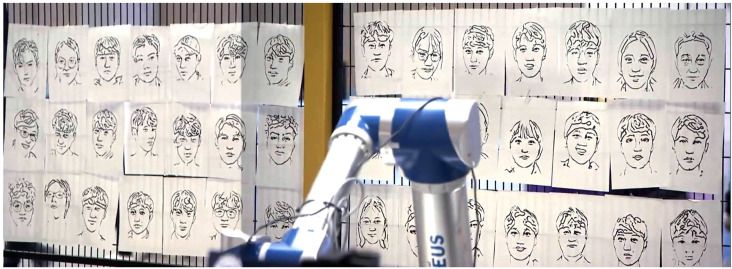
Portrait sketches of some volunteers participated in the RoboWorld exhibition.

**Figure 15:**
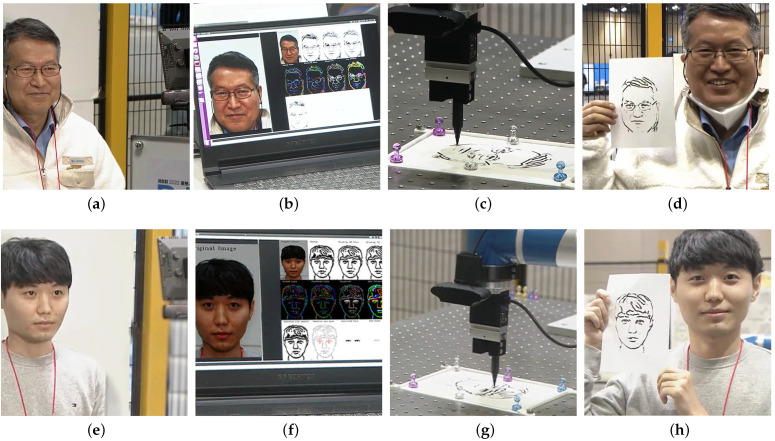
Two volunteers use our proposed system to create personalized portraits. The process involves (**a**,**e**) capturing an image; (**b**,**f**) processing it; (**c**,**g**) starting the drawing; (**d**,**h**) resulting in a visually appealing portrait.

In November 2022, the fully tested system was showcased at the RoboWorld exhibition, as reported in [36]. During this event, we distributed surveys to the volunteers who experienced the system firsthand, seeking their valuable feedback. The system demonstrated its remarkable capability by skilfully drawing portraits of over 40 volunteers, showcasing its efficiency in generating high-quality portrait sketches. As depicted in Figure 14 and Figure 15, the volunteers’ responses reflected an exceptional 95% satisfaction rate, providing clear evidence of the system’s effectiveness and reliability. Furthermore, the system’s outstanding performance earned it the prestigious Korean Intellectual Property Office Award at the competition. This recognition and acclaim highlight the system’s tremendous potential for practical application in various settings, including galleries, studios, and educational institutions. The success of the system at the RoboWorld exhibition not only underscores its viability as a valuable tool for producing top-tier portrait sketches but also paves the way for further development and advancements in the field of robotic art.

## 6. Conclusions

The proposed system for robotic portrait drawing, which combines machine learning techniques such as CycleGAN with morphological transformations and path optimization, has been shown to be highly effective at generating visually appealing and accurate sketches in a rapid and efficient manner. Real-world experiments at the RoboWorld exhibition in November 2022 demonstrated the effectiveness, stability, robustness, and flexibility of the proposed system. Our system drew portraits of over 40 volunteers, achieving a high satisfaction rate of 95% among the participants. The success of our system at the exhibition was recognized with the Korean Intellectual Property Office Award. The results suggest that the proposed system has great potential for practical application in a range of settings, including galleries, studios, and educational institutions.

The high satisfaction rate of 95% among participants indicates the effectiveness and reliability of the system in producing high-quality portrait sketches in a time-efficient manner. The proposed system represents a significant advancement in the field of robotic portrait drawing due to its ability to generate visually appealing and accurate sketches in a rapid and efficient manner. The real-world experiments at the RoboWorld exhibition have demonstrated the effectiveness, stability, robustness, and flexibility of the proposed approach, with an average drawing time of 80 s per portrait.

In summary, the proposed system has demonstrated its capability to generate visually appealing and accurate sketches in a rapid and efficient manner, making it a significant advancement in the field of robotic portrait drawing. The high satisfaction rate achieved among the participants at the RoboWorld exhibition suggests that the proposed system has great potential for practical application in various settings. This research presents a promising direction for the development of robotics and machine learning technologies in the field of portrait drawing.

Furthermore, investigating the adaptability of the proposed system to different artistic styles and mediums, such as watercolor or charcoal, would broaden its applicability and artistic versatility. This could involve exploring different data representations, training methodologies, or incorporating style transfer techniques to allow users to customize the output according to their artistic preferences.

## Figures and Tables

**Table 1:** Comparison of other research papers which utilize pens, pencils, or markers.

	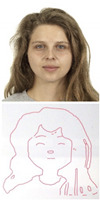	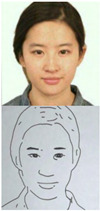	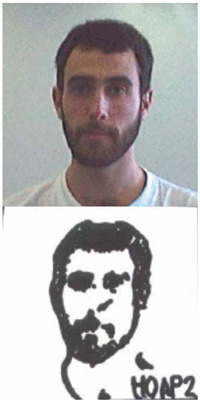	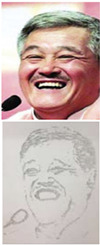	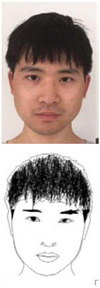	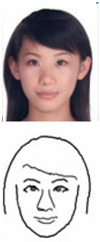	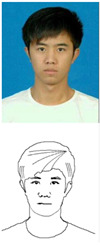	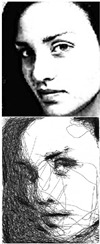
	[25]	[29]	[10]	[26]	[30]	[31]	[32]	[27]
Drawing Time	43.2 s	2 min	2 min	4 min	4–6 min	4–6 min	Not Discussed	Not Discussed
Robotic Arm	UR5 6 DOF	3 DOF	4 DOF	2 DOF	3 DOF	Pica 7 DOF	Not Discussed	Not Discussed
Drawing Tool	Marker	Pencil	Pen	Pencil	Pencil	Pencil	Pencil	Pen
		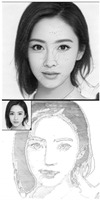		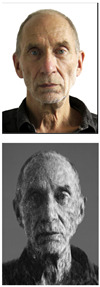		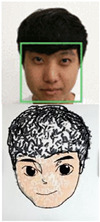		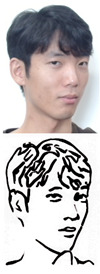
		[28]		[23]		[24]		Ours
Drawing Time		30 min		17 h		1 to 2 h		80 s
Robotic Arm		YASKAWA GP7 6 DOF		Reis Robotics RV20-6 6 DOF		7 DOF		ZEUS 6 DOF
Drawing Tool		Chinese calligraphy pen		Paint Brush		Paint brush		Chinese calligraphy pen

**Table 2:** Summarized specs of the MSI laptop used as the controller server.

CPU	GPU	RAM
intel Core i7-8750H	NVIDIA RTX 2080 8 GB	16 GB

**Table 3:** Summarized time consumption for each module on average.

Portrait Generation	25 ms
Sketch Generation	7 s
Drawing Motion Generation	55 ms
Robot Motion Control	80 s
